# “Diabetic striatopathy”: clinical presentations, controversy, pathogenesis, treatments, and outcomes

**DOI:** 10.1038/s41598-020-58555-w

**Published:** 2020-01-31

**Authors:** Choon-Bing Chua, Cheuk-Kwan Sun, Chih-Wei Hsu, Yi-Cheng Tai, Chih-Yu Liang, I-Ting Tsai

**Affiliations:** 1 Department of Emergency Medicine, E-Da Hospital, Kaohsiung City, Taiwan; 2 College of Medicine, I-Shou University, Kaohsiung City, Taiwan; 3 Department of Neurology, E-Da Hospital, I-Shou University, Kaohsiung City, Taiwan

**Keywords:** Endocrinology, Neurology, Signs and symptoms

## Abstract

Diabetic striatopathy (DS) is a rare medical condition with ambiguous nomenclature. We searched PubMed database from 1992 to 2018 for articles describing hyperglycemia associated with chorea/ballism and/or neuroimages of striatal abnormalities. Descriptive analysis was performed on demographic/clinical characteristics, locations of striatal abnormalities on neuroimages, pathology findings, treatment strategies, and outcomes. In total, 176 patients (male:female = 1:1.7) were identified from 72 articles with mean age 67.6 ± 15.9 (range, 8–92). Among them, 96.6% had type 2 DM with 17% being newly diagnosed. Average blood glucose and glycated hemoglobin concentrations were 414 mg/dL and 13.1%, respectively. Most patients (88.1%) presented with hemichorea/hemiballism. Isolated putamen and combined putamen-caudate nucleus involvements were most common on neuroimaging studies with discrepancies between CT and MRI findings in about one-sixth of patients. Unilateral arm-leg combination was the most frequent with bilateral chorea in 9.7% of patients. Chorea and imaging anomalies did not appear concomitantly in one-tenth of patients. Successful treatment rates of chorea with glucose-control-only and additional anti-chorea medications were 25.7% and 76.2%, respectively, with an overall recurrence rate being 18.2%. The most commonly used anti-chorea drug was haloperidol. To date, four out of six pathological studies revealed evidence of hemorrhage as a probable pathogenesis.

## Introduction

The term “diabetic striatopathy” (DS), also known as “hyperglycemic non-ketotic hemichorea/hemiballism”^1,2^, “chorea/hemichorea associated with non-ketotic hyperglycemia”^3–6^, “ diabetic hemiballism/hemichorea”^7^, or “chorea, hyperglycemia, basal ganglia syndrome”^8^, was first used to describe a relatively uncommon hyperglycemic condition associated with chorea/ballism and unique reversible abnormality of basal ganglia on computed tomography (CT) or/and magnetic resonance imaging (MRI)^9^. Both chorea and ballism refer to random, uncontrollable, involuntary, jerking motions with ballism typically being more proximal and of larger amplitude than chorea. The prevalence of DS has been reported to be 1 in 100,000^10^, which is believed to be underestimated because most physicians are not familiar with the condition which could be misdiagnosed as common intracerebral hemorrhage^11^. The disorder has been documented to occur predominantly in elderly females with type 2 diabetes mellitus (DM). Although previous sporadic case studies proposed that correction of hyperglycemia usually resulted in complete or partial resolution of chorea as well as striatal abnormalities on neuroimaging studies^1^, additional anti-chorea drugs might be needed in patients unresponsive to strict glucose control^12^. Not only does the associated chorea substantially impair the normal daily functions of those afflicted with the disease but the lack of well-established guidelines for treatment may also lead to life-threatening complications. For instance, the administration of deep sedation may be associated with respiratory failure and even mortality^13^.

Due to its rarity, there were only a few larger studies focusing on its incidence, demographic characteristics, symptom correlation, and locations of striatal abnormalities of the condition^5,14^. One meta-analysis investigated 53 patients^5^, while the other study analyzed 20 patients^14^. Nevertheless, the discrepancy between symptom and neuroimaging presentations, comparison between CT and MRI findings, recovery time of chorea, resolution time of neuroimaging anomalies, the effectiveness of different treatment strategies, and the incidence of symptom recurrence as well as the variations in pathological features with disease progression were not explored. Therefore, the purpose of this study was to address this clinically uncommon but important condition through systemically reviewing available literature.

## Materials and Methods

### Literature search strategy and data extraction

We conducted a literature search on the PubMed database using the keywords “diabetic striatopathy”, “hyperglycemic hemichorea/hemiballism”, “hyperglycemic chorea/ballism”, “nonketotic hyperglycemia” combined with “dyskinesia/hemidystonia” or “involuntary movement”, “basal ganglia syndrome”, “striatal anomaly”, “striatal hyperintensity”, and “T1-weighted hyperintensity” between 1992 to 2018. Original articles, case series, case reports and letters that documented specific imaging findings on CT and MRI with or without chorea related to hyperglycemic status published in English or Chinese languages were included in our study. Case reports with results of pathological analysis and images of DS on CT or/and MRI were also included. Each reported case was checked meticulously to avoid repetition. Studies with suspicious repeated description of the same patient population (defined as publications with overlapping study periods from the same institution), those without mentioning striatal locations in describing the neuroimaging findings, those without detailed information on treatment, and/or non-English or non-Chinese literature were excluded from the present study.

The parameters extracted from the selected studies included reporting countries, baseline demographics, medical information, duration of chorea, location of striatal abnormality (e.g., caudate nucleus, putamen or/and globus pallidus), treatment strategies, outcomes, and the duration for the striatal abnormalities to resolve on neuroimaging follow-ups.

### Definitions

In the present study, diabetic striatopathy (DS) was defined as a hyperglycemic condition associated with both or either one of the two following conditions: (1) chorea/ballism; (2) striatal hyperdensity on CT or hyperintensity on T1-weighted MRI as previously reported^9,12,15^.

Three anatomical regions were used in the description of the locations of neuroimaging anomalies in the basal ganglia, namely, caudate nucleus, putamen, and globus pallidus. The terms “mismatch” and “incompatibility” were used in this study to describe the discrepancies between non-enhanced CT and T1-weighted MRI findings. While mismatch between CT and MRI results was defined as the complete absence of anomaly in basal ganglia on one but not the other study, incompatibility referred to the difference in locations of striatal anomalies between the two imaging modalities.

In terms of symptom presentation, we documented chorea involvement in four main body regions: face, trunk, arm, and leg. Furthermore, pre-treatment interval of chorea was defined as the estimated duration between the onset of chorea and treatment, while recovery time referred to the time period from the initiation of treatment to complete resolution of chorea. Regarding treatment outcomes, successful treatment (i.e., complete response) referred to patients with documented complete resolution of chorea or residual minor chorea after treatment, whilst partial response indicated those with documented partial or moderate-degree resolution of chorea following treatment in the present study. For determining the incidence of chorea recurrence, only patients with a minimum of one-month follow-up were included. For comparison between neuroimaging anomalies and clinical manifestation of chorea, the term “inconsistency” represented the condition in which the clinical presentation of chorea did not accompany striatal abnormality on neuroimaging study or vice versa. Ineffective medical treatment against chorea was defined as no response or only slight response after drug administration based on documentation in the reports. In addition, for interpretation of pathological findings, we defined time-delay as the estimated time interval between positive neuroimaging findings and biopsy/autopsy.

### Statistical analysis

A database on patient’s information was created using the Excel® program in which descriptive analysis was performed with categorical variables expressed as percentage and continuous variables shown as means with standard deviation. Non-normally distributed variables (i.e., durations) were shown as medians and 95% confidence intervals (CIs). Fisher’s exact test was used to determine the significance of correlation between CT and MRI findings as well as the recurrence rate of glucose-control-only group and that of additional anti-chorea medication group. A probability value of less than 0.05 was considered statistically significant.

## Results

### Reporting countries and demographic characteristics

**Figure 1:**
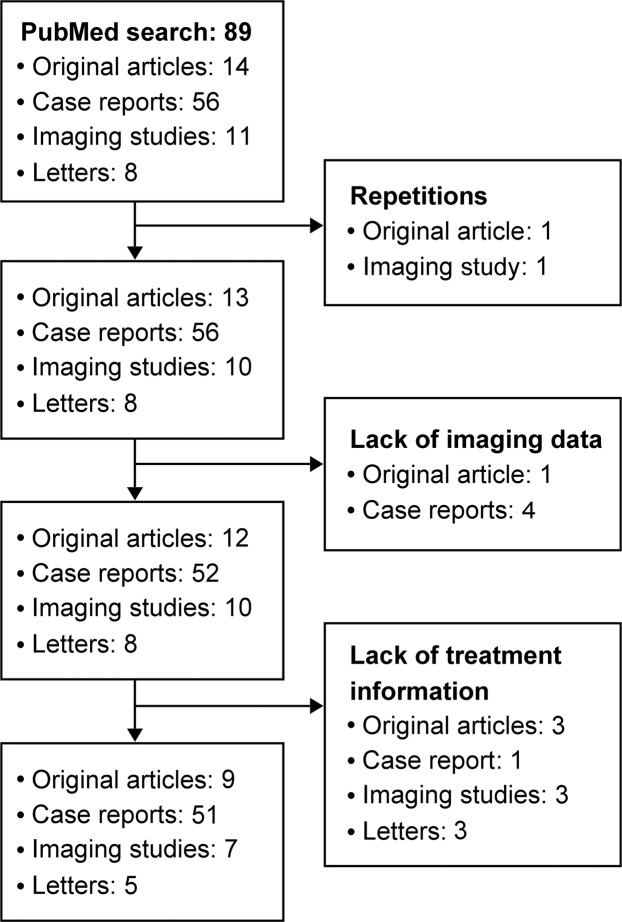
Selection of eligible literature for the present study.

Of the 89 articles retrieved from the PubMed database using the appropriate keywords, 17 were excluded because of repetitions in case description, or lack of information on neuroimaging or detailed treatment strategies (Fig. 1). As a result, 72 articles involving 176 patients were included in the present study^1–9,11–13,15–74^. Countries most frequently reporting the condition were in the order of Taiwan (world population: 0.31%)^17,36,41,54,75^, Japan (1.65%)^15,19,28,40,70,73^, China (18.65%)^3,4,6,37,43,58^, South Korea (0.67%)^5,7,38,55,59,67,71,72^ and USA (4.27%)^30,49,56^. The disorder was also sporadically described in studies from Australia (0.33%)^47,52^, Brazil (2.75%)^45,50^, Canada (0.49%)^16,48^, France (0.85%)^31^, India (17.75%)^12,24,29,44,51,60,65^, Italy (0.79%)^53,61,69^, Malaysia (0.42%)^42^, Nigeria (2.64%)^65^, Poland (0.50%)^57^, Portugal (0.13%)^64^, Romania (0.25%)^62^, Saudi Arabia (0.45%)^27^, Sri Lanka (0.28%)^1^, Switzerland (0.11%)^26^, Tanzania (0.76%)^23^ and Turkey (1.08%)^32^. Altogether, Asia contributed 71.6% (126/176) of the reported cases, followed by Europe (8.5%, 15/176) and Americas (4%, 7/176). The mean age of the 176 patients in our study was 67.6 ± 15.9 (range: 8–92) with a male-to-female ratio of 1: 1.7.

### Hyperglycemic status

In our study, the majority of patients had type 2 DM (96.6%, 170/176) and 3.4% (6/176) were diagnosed as having type 1 DM. Similarly, of the 30 (17%, 30/176) patients with documented newly diagnosed DM, 29 (96.7%) were diagnosed with type 2 DM. In patients with available laboratory information, the average levels of blood glucose and glycated hemoglobin were 414 mg/dL and 13.1% respectively. In addition, of the 71 patients undergoing analysis of ketone bodies, the majority (81.7%, 58/71) were negative for ketone bodies in their blood or urine specimens.

### Involvement of body regions in DS

Regarding clinical presentations, the large majority of patients (172 out of 176 included in this study, 97.7%) presented with chorea or hemichorea. Of the 149 patients with documented areas of involvement, the most frequently affected were arm-leg (58.6%, 85/145) and arm-leg-face (22.1%, 32/145) combinations, followed by isolated arm (13.1%, 19/145), arm-leg-trunk (2.8%, 4/145), arm-face (2.1%, 3/145), arm-leg-face-trunk (0.7%, 1/145), and facial (0.7%, 1/145) involvements. The ratio of left-sided and right-sided involvement is 1.2: 1. Bilateral involvement was noted in 9.7% of patients (17/176).

### Locations of striatal abnormalities

Of the three striatal regions, the most commonly involved in the 126 patients with documented CT findings with or without concomitant anomalies in the other two regions was putamen (78.6%, 99/126), followed by caudate nucleus (47.6%, 60/126), and globus pallidus (27.8%, 35/126). The results were consistent with those of MRI studies in 153 patients showing the frequency of involvement in the order of putamen (94.1%, %, 144/153), caudate nucleus (64/153), and globus pallidus (43/153). The prevalence of striatal anomalies on imaging studies in different locations as either isolated lesions or combinations are shown in Table 1. The prevalence of involvement of one, two, and all three regions was 39% (64/164), 34.8% (57/164), and 26.2% (43/164), respectively. The two regions most commonly reported to show concomitant involvement on both imaging modalities were caudate nucleus and putamen (Table 1). Bilateral striatal involvement was noted in 17 out of the 176 patients (9.7%) undergoing both CT and MRI studies.

**Table 1:** Summary of locations of striatal abnormalities.

Location\Imaging modality	CT (n = 126)	MRI (n = 153)
CN	1	2
CN + P	29	26
CN + GP + P	30	36
GP	0	0
GP + CN	0	0
GP + P	5	15
P	35	67
No anomaly	26	7

CN, caudate nucleus; CT, computed tomography; GP, globus pallidus; MRI, magnetic resonance imaging; n, number of patients; P, putamen.

### Mismatch and incompatibility between CT and MRI findings

**Figure 2:**
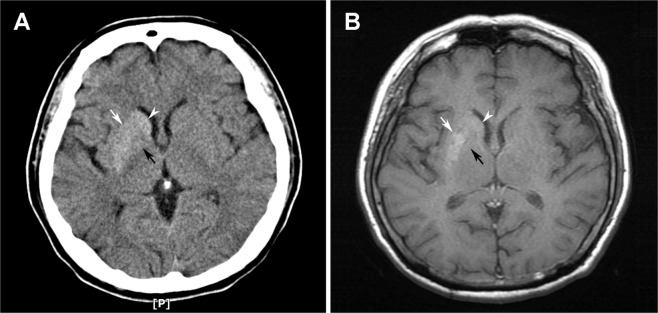
Typical images from (**A**) computed tomography (CT) and (**B**) T1-weighted magnetic resonance imaging (MRI) showing striatal anomalies of putamen (white arrows) and caudate nucleus (white arrowheads). Note the hyperdensity over globus pallidus on CT image but lack of hyperintensity on MRI (black arrows), defined as “incompatibility” in the present study.

Among the 103 patients with available information on both CT and MRI, Fisher’s exact test demonstrated significant correlations between findings from the two imaging modalities (*p* < 0.01). Nevertheless, the mismatch rate was 17.5% (18/103). MRI demonstrated striatal anomalies in four out of the seven patients who exhibited negative findings on CT scans, whereas there was no positive finding in CT scans in patients who displayed negative findings on MRI. Taking into account the difference in locations of striatal anomalies between CT and MRI, the incompatibility rate was 14.6% (15/103). Typical neuroimaging presentations of incompatibility in a patient with diagnosis of DS are shown in Fig. 2.

### Changes in striatal anomalies on CT and MRI follow-ups

The median duration of imaging follow-ups in studies with available information was 120 days (95% CI: 145.4–222.2) (n = 67). Complete or partial resolution of striatal anomalies on imaging studies (i.e., CT or/and MRI) was noted in all of the studies. The median duration of discernible resolution on CT was 24 days (95% CI: 13.8–49.1) (n = 9) in comparison with 120 days **(**95% CI: 79.3–268.5) (n = 18) for MRI studies, while the median complete resolution time of striatal hyperdensities on CT scan was 60 days (95% CI: 57.9–130.6) (n = 25) compared to that of hyperintensities on T1-weighted MRI (i.e., 180 days, 95% CI: 193.1–304.1, n = 29). The shortest resolution time in this study was 10 days on CT compared to 60 days on MRI during follow-ups. Although increase in signal intensity of striatal abnormalities on MRI compared to that of their initial images was reported to be noticeable in 21 patients after a median of 90 days (95% CI: 64.8–122.4) following their hospital discharge, all of their striatal anomalies resolved on subsequent imaging follow-ups.

### Sensitivity of CT and MRI to detecting DS-associated chorea and inconsistency between symptoms and neuroimaging studies

Of all the 176 patients included in the current study, the large majority presented with chorea (97.7%, 172/176). In terms of sensitivity of the two imaging modalities in detecting DS with chorea, the current study showed a sensitivity of 95.33% and 78.86% for MRI and CT, respectively. The inconsistency rate of CT scan (n = 126) and MRI (n = 153) was 20.6% (26/126) and 4.6% (7/153), respectively. The overall inconsistency rate (i.e., chorea with negative imaging finding on one or both imaging modalities or vice versa) was 9% (16/176) which included 12 patients presenting with chorea without striatal involvement on imaging studies (6.8%, 12/176) and four patients showing striatal involvement without the clinical manifestation of chorea (2.3%, 4/176).

### Treatment with glucose control only versus addition of anti-chorea medications and outcomes and alternative therapeutic approaches

**Figure 3:**
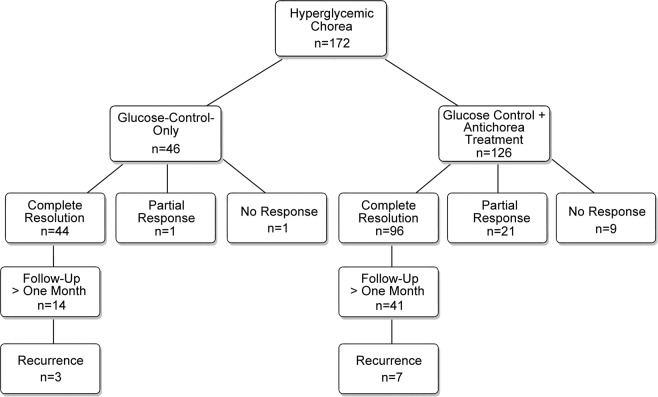
Retrospective review of treatment regimens of patients with hyperglycemic chorea.

To investigate the therapeutic effects of glucose-control-only and anti-chorea treatments against chorea and symptom recurrence, the patients were retrospectively divided into two groups according to their treatment outcomes, namely, those receiving glucose control only and those with the addition of anti-chorea medications. In our study, 26.7% (46/172) of patients received only glucose control and 73.3% (126/172) received glucose control with the addition of anti-chorea medications (Fig. 3).

Successful treatment was achieved in 25.6% (44/172) of patients receiving glucose-control-only treatment during hospitalization. There were two other patients showing either partial response (n = 1) or no response (n = 1) who did not receive anti-chorea medication because of loss to follow-up (n = 1) or mortality (n = 1).

**Figure 4:**
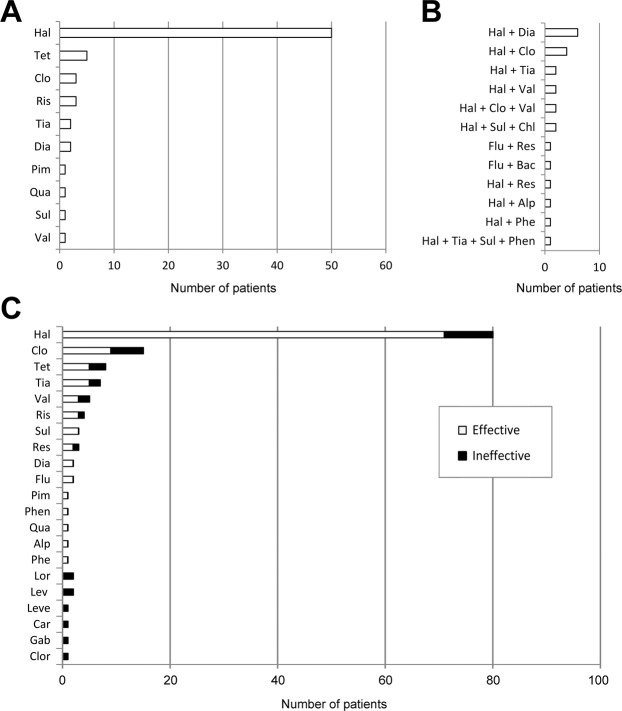
Number of patients with successful anti-chorea treatments using (**A**) monotherapy, and (**B**) combined regimens. (**C**) Overview of frequency of use of individual anti-chorea agent and effectiveness (either as monotherapy or part of combined regimens). Alp, alprazolam; Bac, baclofen; Car, carbamazepine; Chl, chlorpromazine; Clo, clonazepam; Clor, clorazepate; Dia, diazepam; Flu, fluphenazine; Gab, gabapentin; Hal, haloperidol; Lev, levodopa; Leve, levetiracetam; Lor, lorazepam; Phe, phenytoin; Phen, phenobarbital; Pim, pimozide; Qua, quatiapine; Res, reserpine; Ris, risperidone; Sul, sulpiride; Tet, tetrabenazine; Tia, tiapride; Val, valproate.

There were 126 patients receiving anti-chorea medications. The most commonly used single anti-chorea medication for the treatment of DS was haloperidol (n = 50, 39.7%), followed by tetrabenazine (n = 5, 4.0%), risperidone (n = 3, 2.4%) and clonazepam (n = 3, 2.4%) (Fig. 4A). Of all the combined regimens, the combination of haloperidol and diazepam was the most common (4.8%, 6/126), followed by haloperidol and clonazepam (3.2%, 4/126), and haloperidol and tiapride (1.6%, 2/126) (Fig. 4B). There were 21 patients with partial response and 9 without response, giving a successful treatment rate of additional anti-chorea medications of 76.2% (96/126). The anti-chorea medical regimens and their effectiveness are summarized in Fig. 4C. More aggressive approaches including pallidotomy (n = 1)^16^, ventrolateral thalamotomy (n = 1)^5^, transcranial magnetic stimulation (n = 1)^46^, and globus pallidus internus deep brain stimulation (n = 1)^7^, which were reserved for patients with intractable chorea, also improved their symptoms.

### Correlation of outcome with pre-treatment interval of chorea

There was no significant difference in the median pre-treatment interval of chorea in patients receiving glucose-control-only treatment (4 days, 95% CI: 3.61–8.19, n = 29) compared with that in those with additional anti-chorea agents (4 days, 95% CI: 7.0–18.3, n = 62). Besides, the median pre-treatment interval in patients with successful treatment of chorea (4 days, 95% CI: 6.0–15.28, n = 76) was not significantly different from that of those with partial response and no response (2 days, 95% CI: 3.3–16.0, n = 15).

### Recovery time and recurrence rate of chorea

Of the 44 and 96 patients with complete resolution after receiving glucose-control-only and anti-chorea medical treatments, respectively (Fig. 3), recovery time was reported only in 34 and 57 patients, respectively. Of the 34 patients with documented successful treatment after glucose-control-only therapy, the median recovery time was 2 days (95% CI: −0.6–24.3), whereas the median recovery time of the 57 patients receiving anti-chorea medications with successful treatment was 14 days (95% CI: 21.5–45.8). It was significantly shorter in patients undergoing glucose-control-only treatment than that in those receiving additional anti-chorea medications.

Similarly, of the 44 and 96 patients with complete resolution after receiving glucose-control-only and anti-chorea medical treatments, respectively (Fig. 3), recurrence status was documented only in 14 and 41 patients, respectively, with a follow-up period of over one month. The recurrence rate of the 14 patients in the glucose-treatment-only group was 21.4% (3/14) in comparison with 17.0% (7/41) for the 41 patients receiving additional anti-chorea treatment (Fig. 3), giving an overall recurrence rate of 18.2% (10/55). There was no significant difference in recurrence rate between the two groups.

### Pathological analyses

Of the six reports with available information on pathological analysis, astrocytosis was documented in all studies^9,13,64,73,75,76^. There were four studies describing the presence of macrophage infiltration^9,13,64,73^. One biopsy^9^ and three autopsies^13,64,73^ with time-delay within 33 days demonstrated evidence of hemorrhage with erythrocyte extravasation, focal microhemorrhages, extravascular hemosiderin deposits, and hemosiderin-contained macrophages, respectively. Another stereotactic biopsy performed after a time delay of 60 days showed gliosis, hyalinosis of blood vessels and abundant gemistocytes, which are swollen reactive astrocytes that often appear during acute injury with subsequent gradual shrinkage in size^75^. Other reported pathological findings also include confluent infarction^13^, multiple small foci of tissue necrosis^9,73^, lumen narrowing of arterial wall with fibrosis^64,73^, punctuate calcification in infarct area^13^ and calcium deposits^74^.

## Discussion

Although the disease of “hemiballism” was first described six decades ago^77^, the term “diabetic striatopathy” (DS) was introduced merely a decade ago to denote the condition in which there is a combination of striatal hyperintensity on T1-weighted MRI and contralateral movement disorder in diabetic patients^9^. There was a large time gap before the term being expanded in recent years to also include those presenting with either imaging anomalies^15^ or chorea^12^. Although DS is a well-documented etiology of chorea/ballism, most previous reports on DS did not provide detailed descriptions on the presentations. The occurrence of chorea/ballism is mostly due to dysfunction of basal ganglia and subthalamus^75^. Beside DS, other etiologies of chorea/ballism include cerebrovascular, autoimmune, toxic, malignant and infectious illness^5^. The ambiguity in definition of DS and the different terms used to describe the condition may have led to underestimation of the prevalence of the disease. The present systematic review is the first to address the inconsistency between symptom and neuroimaging presentations, mismatch and incompatibility between CT and MRI findings, resolution time of neuroimaging anomalies, the effectiveness of different treatment strategies, symptom recurrence incidence, and disease progression-associated pathological changes.

To date, there have only been a few original articles in the literature, including two relatively large series. While one study reported 20 patients within four years at one institute^14^, the other described 25 patients within six years at five hospitals^78^. Both reported slight female predominance and susceptibility of the elderly to DS as in the present study. Compared to an earlier meta-analysis on DS investigating only adults with hyperglycemic hemichorea^5^, the current study expanded to childhood and enrolled four patients below 18 years of age with the youngest being eight years old. In addition, though Asian patients were most commonly reported in previous studies, the present study showed that there have been an increasing number of reports from Europe, North and Latin America.

DS typically happened in patients with long-standing poor control of DM which was further confirmed by remarkable elevation of blood glucose and glycated hemoglobin level in our study, and it could even occur after correction of hyperglycemia^17^. Consistently, the majority of DS patients (96.6%) in the current study had type 2 DM including one sixth with newly diagnosed diabetes, suggesting that DS could be the first presentation of DM. Regarding the association between DS and ketosis, a large majority (81.7%) of patients with documented ketone status in the present study were not ketotic, compatible with the term “hyperglycemic non-ketotic hemichorea/hemiballism”. On the other hand, ketosis in the remaining 18.3% patients implied that occurrence of DS is not restricted to non-ketotic patients. The susceptibility of DS to non-ketotic hyperglycemic condition may arise from the underlying pathophysiology of chorea. In non-ketotic hyperglycemic status, brain metabolism shifts to the alternative anaerobic pathway in Krebs cycle that leads to rapid depletion of gamma-aminobutyric acid (GABA), resulting in disinhibition of subthalamus and basal ganglia that causes hyperkinetic movements in DS patients. At the other end of the spectrum, in ketosis, GABA can be resynthesized by using acetoacetate produced in the liver to prevent its reduction, thereby explaining the less common occurrence of DS in diabetic ketoacidosis^5,12^.

The results of the current study showed that chorea of DS mostly involved unilateral limbs with only 9.7% of bilateral involvement. Before onset of chorea, there were some prodrome symptoms including chest pain^21^, shoulder pain^24^, headache^40^, gait imbalance^49^, hemiparesis^49^, lethargy^61^, stiffness^31^, vertigo^70^, dizziness^17,34^, confusion^69^ and coma^11^. The presentations of the involuntary movements in DS varied among patients. They could start abruptly or insidiously from low to high amplitude, and manifest intermittently or continuously. Cases with chorea progressing from upper to lower extremities^6,66,70^ were more common than lower to upper extremities^19^. In addition, most patients with chorea worsened during nervousness and disappeared after sleep. Only two reported cases showed no suppression of chorea during sleep^23,30^. Although the majority of DS manifested with chorea/hemichorea, there were four patients without chorea who presented with conscious disturbance^15,66^, seizure^66^, limb weakness^54^, dysarthria and dysphagia^54^.

Compatible with the findings of previous reports^5^, the most common pattern of striatal anomalies of DS was isolated putamen involvement, followed by combined caudate nucleus-putamen involvement. Concomitant occurrence of anomalies in all three striatal components was also noted in over one-fourth of all cases (Table 1). The reason for striatal vulnerability to DS remains unclear. In terms of body regions affected, despite the highest frequency of extremity involvement in the order of arm-leg, arm-leg-face, and isolated arm, there were two reported cases with isolated facial hemichorea presenting with oral dyskinesia and grimacing^70,79^. No significant association was noted between the body region involved and the location of striatal anomaly.

CT and MRI were the two commonest imaging modalities to detect striatal anomalies of DS. Despite the highly significant correlations between their findings, there was around one sixth of mismatch (17.5%) and incompatibility (14.6%) between results of CT and MRI in our study. With regard to mismatch between findings from the two imaging modalities, the current study revealed that MRI is more sensitive to the detection of DS-associated striatal anomalies as it demonstrated striatal lesions in patients who exhibited negative CT results, whilst there was no positive finding on CT scans in patients showing no abnormality in all three regions of basal ganglia on MRI. However, our results were only based on the included studies. Considering the possibility that CT and MRI may detect anomaly in different regions of basal ganglia (i.e., incompatibility, Fig. 2), CT is still indicated for patients with negative MRI findings.

In view of the correlation between imaging findings and clinical symptoms, the present study not only demonstrated a higher sensitivity of MRI than that of CT to the detection of hyperglycemic chorea (95% vs. 79%, respectively) but also revealed a much lower inconsistency rate (4.6%) compared with that of CT (20.6%). Therefore, the findings suggested that MRI may show better correlation with the presence of chorea compared with CT. In addition, the follow-up striatal abnormalities of CT images seemed to resolve faster than those of MRI. The shortest follow-up resolution time on CT was 10 days compared to 60 days on MRI in our study, implying that MRI may be a more accurate tool for tracing the resolution of striatal anomalies in follow-up studies. Furthermore, the current study also revealed an increase in signal intensity of the striatal hyperintensity during serial follow-ups on MRI which was not shown on CT scans, indicating that striatal hyperintensity might reach a maximum level on an average of 3 months (93.6 days) after which the intensity began to decline.

Although a wide variety of mechanism can lead to striatal hyperintensity on T1-weighted MRI including hypertensive hemorrhage, calcification, genetic diseases (e.g., Tay-Sachs disease, tuberous sclerosis, neurofibromatosis, Fahr disease), metabolic disorders (e.g., Wilson’s disease, hypoglycemic coma, chronic hepatic encephalopathy), toxicity (e.g., manganese toxicity, carbon monoxide poisoning), and brain ischemia (e.g., lenticulostriate infarction, postcardiac arrest encephalopathy), the striatal lesions are mostly bilateral except in the case of hypertensive hemorrhage^21^. One of the distinctive features of DS-associated striatal anomaly to differentiate it from hypertensive hemorrhage is the absence of mass effect and the sparing of the internal capsule^65^. This unique imaging finding, when combined with hyperglycemia and the presence of chorea as noted in the majority of patients in the present study, is pathognomonic of the condition.

To date, there have been four hypotheses to explain the pathogenesis underlying the observed striatal anomalies on neuroimages, namely petechial hemorrhage^64^, mineral deposition (i.e., calcium or magnesium)^13^, myelin destruction^56^, and infarction with astrocytosis^13,75^. Researchers initially attributed striatal anomalies to petechial hemorrhage based on the observation of hyperdensity on CT and hyperintensity on MRI strongly suggestive of the presence of hemorrhage and methemoglobin, respectively^80^. Other previous studies utilized different imaging modalities in an attempt to elucidate cellular function and perfusion status of the affected region. For instance, studies from diffusion-weighted MRI^72^ and susceptibility weighted MRI^47^ suggested hyperviscosity with cytotoxic edema and deposition of paramagnetic material, respectively. Positron-emission tomography (PET) studies demonstrated marked decrease in glucose metabolism of the lesioned basal ganglia^17^, while single-photon emission CT (SPECT) revealed mostly hypoperfusion in the corresponding region^71^. A recent study using magnetic resonance angiography revealed oozing around the basal ganglia lesion^15^.

Unlike radiological studies, pathological analysis could shed direct light on the pathogenesis of DS-associated striatal lesions. Although myelin destruction may appear as hyperintensity on T1-weighted MRI, there was no evidence suggesting its presence according to the six available pathology reports. However, reactive astrocytosis documented in five pathology reports^9,13,64,73,76^ and abundant gemistocytes in another biopsy^75^ may partially explain the striatal hyperintensity on T1-weighted MRI but not hyperdensity on CT. On the other hand, there were only two pathology reports showing some calcium deposits^73^ or punctuate calcification^13^ which, however, could not account for the observed resolution on subsequent neuroimaging studies. In contrast, microvascular hemorrhage may be a probability based on the findings of four pathological analyses showing hemosiderin-contained macrophages^73^, microhemorrhage^13^, extravascular hemosiderin deposits^64^, and erythrocytes extravasation^9^, respectively.

The mainstay of DS treatment is control of hyperglycemia with proper hydration to correct the underlying metabolic imbalance^12^. The present study revealed that although chorea could be successfully treated with glucose control only in one-fourth of the patients, the majority needed additional anti-chorea medications for symptom control. There are four main categories of anti-chorea medications, namely antipsychotics, GABA-receptor agonists, selective serotonin reuptake inhibitors and dopamine-depleting agents^12^. The current study showed that haloperidol was the most common monotherapeutic agent against DS-associated chorea, followed by tetrabenazine, risperidone and clonazepam. Other anti-chorea medications included tiapride, quatiapine, pimozide, diazepam and vaproate. Combined regimens were also sporadically documented. For patients with intractable chorea, more aggressive approaches may be indicated. The present study demonstrated a high degree of effectiveness of anti-chorea medications for chorea relief after failure of glucose control for resolving symptom.

The lack of significant difference in treatment intervals both between the insulin-control-only group and the additional anti-chorea medication group as well as between the complete response and poor response groups suggested that the timing of treatment intervention may not be a critical contributor to patient outcomes which appeared to depend more on the severity of the underlying condition. Therefore, the significantly shorter recovery time of the insulin-treatment-only group than that of the additional anti-chorea medication group may reflect a less severe disease in the former compared with that in the latter. Taking into account the possibility of recurrent chorea even after the resolution of striatal anomaly^40^, the relatively high overall recurrence rate of close to 20% highlights the need for regular follow-ups regardless of the neuroimaging findings.

There were several limitations to the present study. First, the relatively small number of studies precluded reliable comparison and elucidation of the effectiveness of different medications and alternative treatment approaches. Second, because the time between glucose control failure in chorea relief and the implementation of anti-chorea medications was not mentioned in most studies, the differences in severity of symptoms between the two groups and the effectiveness of the two treatment strategies could not be compared. Third, most included studies merely reported the presence of ketone bodies in urine and/or blood as a diagnostic tool for ketosis without mentioning the exact level so that the severity of ketosis could not be determined. Fourth, the limited number of pathology reports available could not shed enough light for understanding DS-induced striatal pathogenesis. Fifth, detailed information on recovery time was only available in some but not all of the studies. Finally, accurate estimation of resolution time of striatal lesions depended on the completeness of follow-ups which could not be achieved in some studies.

## Conclusions

In conclusion, in an attempt to comprehensively investigate “diabetic striatopathy” taking into account the use of different terms for the disorder in literature, the present study analyzed the manifestations, course and treatments as well as possible pathogenesis of the disease. The results demonstrated that, although chorea could be successfully treated with glucose control only in one-fourth of the patients, the majority required additional anti-chorea medications for symptom control. Regarding imaging presentations, isolated putamen and combined putamen-caudate nucleus involvements were most common. Discrepancies between CT and MRI findings were noted in about one-sixth of the patients, whilst chorea and imaging anomalies did not appear concomitantly in around one-tenth of patients. Most cases showed resolution of imaging anomalies about three months on CT and over eight months on MRI. Haloperidol was most frequently used for chorea treatment which was successful in most cases. Moreover, pre-treatment intervals for chorea had no significant impact on patients’ recovery time which was shorter for those with glucose control only (i.e., less than two weeks) than that for those requiring additional anti-chorea medications (i.e., one month). Furthermore, recurrence still occurred in close to one-fifth of documented patients even after resolution of striatal anomalies.

## Data Availability

The data that support the fndings of this study are available from the corresponding author (Prof. Cheuk-Kwan Sun) upon request (lawrence.c.k.sun@gmail.com).
